# Patient Satisfaction and Understanding of Moderate Sedation During Endoscopy

**DOI:** 10.7759/cureus.7693

**Published:** 2020-04-16

**Authors:** Pujitha Kudaravalli, Sana Riaz, Sheikh A Saleem, Venkata Satish Pendela, Praise Njoku Austin, Debra A Farenga, Dhruv Lowe, Muhammad Osman Arif

**Affiliations:** 1 Internal Medicine, State University of New York (SUNY) Upstate Medical University, Syracuse, USA; 2 Gastroenterology, State University of New York (SUNY) Upstate Medical University, Syracuse, USA; 3 Internal Medicine, Rochester General Hospital, Rochester, USA; 4 Internal Medicine, Syracuse VA Medical Center, Syracuse, USA

**Keywords:** moderate sedation, patient comfort, patients satisfaction, patient counseling

## Abstract

Millions of endoscopic procedures are performed in the US every year and the use of procedural sedation analgesia (PSA) is increasing with more procedures being performed outside the operating theater and gaining popularity due to reduced costs. Patients having endoscopic procedures usually expect that they would be deeply sedated during the procedure despite verbal counseling during pre-procedure clinic visits and are often dissatisfied with procedural awareness and discomfort. In order to better educate patients, written supplementary reading material was provided to the patients, which stated a clear goal of comfort during the procedure rather than deep sedation. The results showed that the written supplementary material did not improve the patient’s understanding or remembrance of being counseled about moderate sedation. We emphasize that there is no substitute for a physician’s repetitive verbal counseling.

## Introduction

Millions of endoscopic procedures are performed in the US every year. These are usually done under moderate sedation or monitored anesthesia care (MAC) and occasionally under general anesthesia. The use of anesthesia services has been seen to become increasingly prevalent, adding to a significant increase in the cost of these procedures. Patients having endoscopic procedures usually expect that they would be deeply sedated during the procedure despite verbal counseling during pre-procedure clinic visits and are often dissatisfied with procedural awareness and discomfort. In order to better educate patients about moderate sedation, we provided written education materials on moderate sedation to the patients in addition to verbal counseling. The written material had information about moderate sedation and a clear goal of comfort during the procedure rather than deep sedation. We aimed to evaluate if this additional modality of patient education improved patient understanding and satisfaction.

## Materials and methods

This is a prospective study. A total of 637 patients were included in the study. Patients greater than 18 years of age who were able to consent and were suitable to undergo outpatient esophagogastroduodenoscopy (EGD) and colonoscopy under moderate sedation were included in the study. Non-English-speaking patients were excluded from the study. The study was conducted from March 2019 to July 2019. Four outpatient gastrointestinal (GI) clinic providers gave the written flyer to the patients after extensive verbal counseling. Ten other GI clinic providers counseled the patient only verbally about moderate sedation. All the patients received a questionnaire that included two questions on the day of the endoscopic procedure. The first question stated, "Did your doctor/nurse practitioner explain to you about moderate sedation that is the medications that we are going to use to keep you comfortable during the procedure?" The second question stated, "Do you understand that you might not be deeply asleep during the procedure?" The nurses requesting the patients to fill in the questionnaire were blinded to the form of counseling and were not aware of the patients that received supplementary written material in addition to verbal counseling.

Continuous and categorical data were analyzed using t-tests and chi-square tests, respectively. In addition, logistic regression was used to compute the odds ratio (OR) for patients remembering the explanation of the procedure and understanding expressed by the patient regarding the sedation procedure between the two groups. The statistical significance was set at p<0.05. To compare the overall group results further, we performed logistical regression using age and sex as independent variables. Data analysis was performed using SPSS for Mac statistical software 26.0 (SPSS Inc., Chicago, IL).

## Results

A total of 637 patients were included in the study. Group 1 includes patients who received verbal counseling supplemented by written information and Group 2 included patients who only received verbal counseling. The mean age in Group 1 was 50.5 and in Group 2, it was 54.3. The results showed no significant difference between the groups for patients remembering the explanation of the procedure sedation: OR 1.003 (95% CI; 0.992 - 1.014, p-value = 0.63) or expressing an understanding of the sedation procedure: OR 1.008 (95% CI; 0.997 - 1.019, p-value = 0.15). Providing written information in addition to verbal counseling did not improve patient understanding of moderate sedation, as seen in Tables 1-2.

**Table 1:** Baseline patient characteristics The mean age of the patients in study Group 1, that is, patients who received both verbal and written counseling was 50.5, with 47% of the study group being males. In group 2, the mean age was 54.3, with 49% males.

	Group 1 (N = 92)	Group 2 (N = 545)	p-value
Age, year (Mean, SD)	50.5 (11.5)	54.3 (15.2)	0.97
Sex			0.67
Male	43	268	
Female	49	277	

**Table 2:** Patient's memory of counseling and understanding of moderate sedation There was no difference in patients remembering that they were counseled between the two groups. There was also no difference in the number of patients understanding moderate sedation between the two groups.

	Group 1	Group 2	p-value
Patients remembered being explained about moderate sedation			0.48
Yes (n)	54	341	
No (n)	38	204	
Patients expressed understanding moderate sedation			0.09
Yes (n)	55	374	
No (n)	37	171	

## Discussion

Moderate sedation was previously referred to as conscious sedation. It is defined as a drug-induced depression of consciousness during which patients respond purposefully. The use of procedural sedation analgesia (PSA) is increasing with more procedures being performed outside the operating theater and gaining popularity due to reduced costs. The main goals of procedural analgesia are to minimize pain and anxiety, minimizing the patient’s motion during the procedure and maximizing the chances of success of a procedure, and also to return the patient to baseline quickly after a procedure while ensuring that the patient is safe [1]. The guidelines for procedural sedation analgesia are set by several commissions that are followed uniformly throughout a hospital [2-4].

Anxiolytics, analgesics, and dissociative medications are used for moderate sedation. Commonly used medications are midazolam, fentanyl, and meperidine. Naloxone and flumazenil are antidotes that are commonly used as reversing agents. The combination of using benzodiazepine along with opiates is shown to improve patient tolerance either by an additive or a synergetic effect. A study by Wong et al. showed that intravenous sedation with midazolam, alfentanil, or both in combination reduced pain perception and pain recall [5]. On the contrary, a study by Froehlich et al. showed that a combination of midazolam and pethidine does not improve patient tolerance and decrease pain as compared to either drug alone. Verbal amnesia was seen in 60% and visual amnesia in 90% of the patients. In addition, it was noted that the presence of amnesia was not associated with better patient tolerance or less pain. This was noted when a combination of pethidine and midazolam was administered together or alone [6].

A majority of patients undergoing moderate sedation for endoscopy experience discomfort and report remembering the events during the procedure. There are several aspects contributing to patient dissatisfaction. Some of them are high expectations that they will be deeply sedated, increased anxiety, lack of knowledge of moderate sedation and what to expect, history of chronic opioid use, language barriers, and level of literacy.

In our study, 54 of 92 (59%) patients who received verbal counseling supplemented by written information remembered that they were counseled and expressed understanding. Thirty-seven of 92 (40%) neither remembered that they were counseled, nor did they understand the concept of moderate sedation. Interestingly, one patient remembered that he was counseled but did not understand. Among patients who received only verbal counseling, 340 of 545 (62%) patients remembered that they were counseled and expressed understanding while 171 of 545 (31%) neither remembered that they were counseled nor did they understand the concept of moderate sedation. Thirty-five patients of 545 (6%) remembered being counseled but did not understand moderate sedation. Providing written information about moderate sedation during clinic visits did not improve the patient’s understanding of moderate sedation as observed in our study. Most patients get supplemental reading material during their clinic visit explaining different conditions, vaccinations, procedures, and self-care in regards to a particular condition but a study by Davis et al. showed that the average reading comprehension grade should be between 11-14 (college level) for understanding written medical education materials and it is fifth grade in community clinics and tenth grade in private practices [7]. Even if a patient had the ability to interpret written material, it is questionable as to how many patients go through the paperwork handed over to them.

Verbal counseling is the most important means of explaining medical conditions or procedures to patients. Low education levels might be a contributing factor in our clinic population for written supplemental education material not improving the outcome. Patient-provider communication is key in patient education supplemented by other materials. Communication is influenced by many factors such as the patient’s learning style, literacy level, culture, and environment. There is no ideal patient education conversation and it is challenging given the large base of medical terminology and inadvertent use of terms. It is important for the provider to understand the patient’s level of understanding and incorporate demonstrations, diagrams, reinforcement, review, teach-back, and support [8-9].

Patients may need to be counseled multiple times in order for them to remember and understand the information being given to them. In our study, 171 of 545 patients in Group 2 and 37 of 92 patients in Group 1 did not even remember that they were counseled. The "rule of seven," the well-known marketing arcade states that a subject needs to hear a message at least seven times before they register, take an action, or buy. This can be translated into medical education in that a patient may need education at multiple visits, including during the clinic visit, over a telephone encounter a day before the procedure, on the day of the procedure, and so forth.

The strengths of our study included blinding the nurses who distributed the questionnaire to the patients and the prospective nature of the study. Our study was limited by having a smaller number of patients in Group 1 as compared to Group 2.

## Conclusions

Written education materials in addition to verbal counseling on moderate sedation did not help improve patient education and patient satisfaction. Physicians should allocate sufficient time to verbally counsel patients, while taking into account the patient’s level of education, and reinforce the education before the patient undergoes an endoscopic procedure to improve patient satisfaction and outcomes. An understanding of moderate sedation by the patients will set their expectations prior to the procedure and will decrease anxiety. This can improve procedure outcomes in addition to increase patient tolerance of the procedure. This will also enhance patient compliance if/when a repeat procedure is indicated medically.
